# The assessment of the prognosis of musculoskeletal conditions in older adults presenting to general practice: a research protocol

**DOI:** 10.1186/1471-2474-7-84

**Published:** 2006-11-10

**Authors:** Christian D Mallen, George Peat, Elaine Thomas, Simon Wathall, Tracy Whitehurst, Charlotte Clements, Joanne Bailey, Jacqueline Gray, Peter R Croft

**Affiliations:** 1 Primary Care Musculoskeletal Research Centre, Keele University, UK

## Abstract

**Background:**

Musculoskeletal conditions represent a common reason for consulting general practice yet with the exception of low back pain, relatively little is known about the prognosis of these disorders. Recent evidence suggests that common 'generic' factors may be of value when assessing prognosis, irrespective of the location of the pain. This study will test a generic assessment tool used as part of the general practice consultation to determine prognosis of musculoskeletal complaints.

**Methods/Design:**

Older adults (aged 50 years and over) presenting to six general practices with musculoskeletal complaints will be assessed as part of the routine consultation using a generic assessment of prognosis. Participants will receive a self-completion questionnaire at baseline, three, six and 12 months post consultation to gather further data on pain, disability and psychological status. The primary outcome measure is participant's global rating of change.

**Discussion:**

Prognosis is considered to be a fundamental component of scientific medicine yet prognostic research in primary care settings is currently neglected and prognostic enquiry is disappearing from general medical textbooks. This study aims to address this issue by examining the use of generic prognostic factors in a general practice setting.

## Background

Musculoskeletal conditions are a common reason for presenting to primary care where it represents up to 18% of a general practitioner's (GP) workload [1]. Although healthcare systems vary between countries, primary care is generally regarded as the point of first contact for musculoskeletal conditions, and is the setting in which the majority of cases are assessed and managed [2].

Estimating the future course of musculoskeletal conditions is an important consideration in the primary care consultation for patients and health care professionals. An awareness about what is likely to happen in the future allows both patients [3] and professionals [4] to formulate a plan for management. Beyond the individual patient, being able to identify groups at high risk of poor outcome and the factors that may be responsible for the poor prognosis has wider implications for public health initiatives, such as the targeting of obesity as a risk factor for disabling knee pain [5].

However, determining the prognosis of musculoskeletal conditions is difficult. With the notable exception of low back pain, there is currently only a limited amount of information available on the prognosis of other musculoskeletal conditions in primary care [6]. Information on prognostic factors for regional musculoskeletal pain is undoubtedly useful to a clinician and several prognostic indicators consistently emerge from the literature as candidates for a generic assessment of musculoskeletal prognosis. These indicators include pain severity, pain duration, widespread pain, previous pain episode, greater movement restriction, higher levels of anxiety, depression and psychological distress, older age, use of coping strategies, lower social support and disability [7]. The use of these 'generic indicators' to rapidly assess musculoskeletal prognosis in a general practice consultation will be assessed in this study.

This paper outlines the proposed protocol for a study of generic prognostic indicators in older adults consulting their general practitioner with musculoskeletal conditions.

### Objective

The primary objective of this study is to determine the prognostic value of a brief assessment in adults aged 50 years and over presenting to general practice with musculoskeletal conditions.

The specific **aims **of this study are to:

• Develop a brief, practical tool for assessing pain in older people presenting with musculoskeletal conditions in primary care

• Test its feasibility and practicality for use in the general practice consultation and its acceptability to general practitioners

• Determine its ability to predict subsequent outcome

The broad practical and clinically applicable **purposes **of the research are to:

• Provide primary care clinicians with a simple standard method to rapidly identify patients with different prognoses, alongside their usual clinical assessment and judgement

• Encourage primary care clinicians to attend to prognostically relevant features of pain and disability as well as to traditional biomedical characteristics of musculoskeletal pain in older people

• To provide the basis for future research into the usefulness of such pain classification for patient care and treatment selection

## Methods/design

This study has received ethical approval from the Central Cheshire Local Research Ethics Committee (REC Reference number: 06/Q1503/60).

### Design

Prospective observational cohort study in general practice.

### Study base

Older adults (aged 50 years and over) registered with six general practices presenting with an episode of musculoskeletal pain to the general practitioner between September 2006 and March 2007.

### Sampling

All recorded general practice consultations for musculoskeletal pain among adults aged 50 years and over in 6 general practices in Central Cheshire occurring between September 2006 and September 2007 (participants may only be sampled once).

#### Inclusion criteria

• Aged 50 years and over

• Registered with one of the participating practices during the study period

• First Read-coded face-to-face GP consultation for a musculoskeletal condition during the study period (may be first, new episode, or ongoing consultation) (full list of Read codes available from corresponding author on request)

• Provided written informed consent to further contact and medical record review

#### Exclusion criteria

• Red flag pathology – recent trauma likely to be associated with significant injury; acute, red, hot, swollen joint

• Inflammatory arthropathy

• Vulnerable groups – e.g. significant cognitive impairment, dementia, severe/terminal illness

### Recruitment procedures

**Figure 1:**
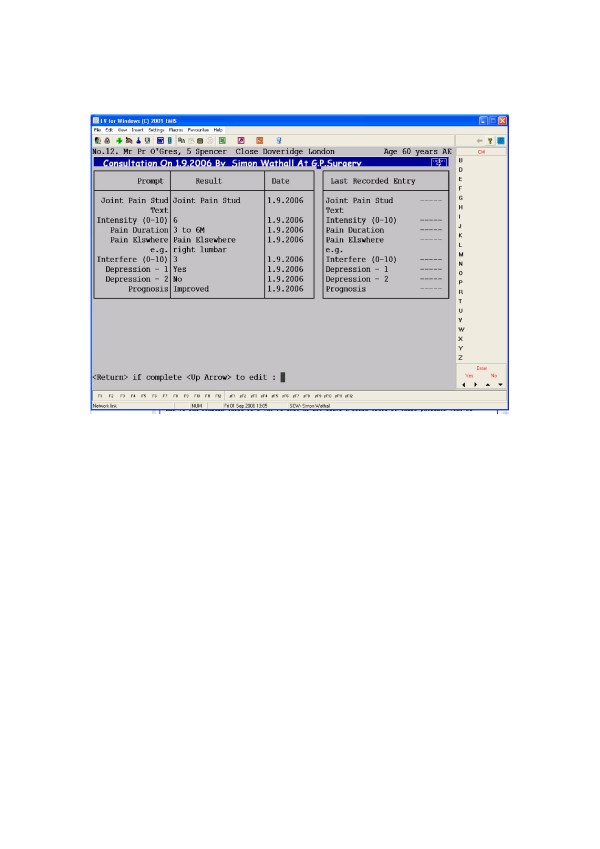
Electronic template.

Potentially eligible participants will be identified in the consultation by the activation of an electronic template when a selected musculoskeletal Read code is entered into the Egton Medical Information System (EMIS). On activation of the electronic template, general practitioners may use their discretion to apply the exclusion criteria as appropriate and exit the template. If not excluded, the electronic template will remind general practitioners of the study and prompt them to record their responses to seven brief questions. The consultation will continue (e.g. with advice, treatment) as per normal. The consultation record of that patient will be electronically "stamped". The template will not be activated if the patient has already had an eligible consultation within the study period, thereby ensuring that patients can only be sampled once. The electronic template is presented in Figure 1. The following questions are asked by the participating GP:

In addition to the six questions given in Table 1, the GP is also asked for their opinion on the likely prognosis for the patient, using the question below:

What do you think the outcome of this patient will be in 6 months time? (response items: completely recovered, much improved, improved, same, worse, much worse).

Weekly electronic searches by members of the Research Network Team will identify all eligible patients in the preceding week with a stamped record. Eligible patients' names and addresses will be downloaded into a secure mailing database. No other information will be accessed unless and until written informed consent to do so is obtained from the patient.

Throughout the entire mailing process, the Research Network team will perform weekly checks for patient deaths and departures, to ensure that patients do not get inappropriately contacted. Participants returning their questionnaire and consent sheet will be logged on the database and no further reminders will be sent.

Eligible patients will be sent a Study Pack from their general practitioner, containing a Patient Information Sheet outlining the study, inviting them to take part, and telling them what they have to do if they would like to take part. The name and contact telephone number of the Principal Investigator will be provided should potential participants have any questions about the study. The Study Pack will contain a Baseline Questionnaire with Consent Form and a stamped addressed envelope.

### Non-responders and non-consenting responders to mailed Study Pack

Non-responders to the mailed Study Pack will be sent a Reminder Postcard at two weeks and a Reminder Letter with repeat Baseline Questionnaire two weeks later. Non-respondents after all three baseline mailings will be assumed not to have consented to take part and will not be contacted again. The mailing database will record non-respondents and non-consenting responders (either by telephone or post) to ensure that patients are not invited to take part more than once during the study period. Participants who consent to be followed up by postal questionnaire but do not want their medical records accessed will receive mailings at three, six and 12 months but will not have their medical record accessed.

### Follow-up of consenting responders to mailed Study Pack

Participants who return their Baseline Questionnaires and provide written informed consent to further contact and to accessing their medical record will be sent Follow-up Questionnaires at 3, 6, and 12 months after their index consultation.

**Figure 2:**
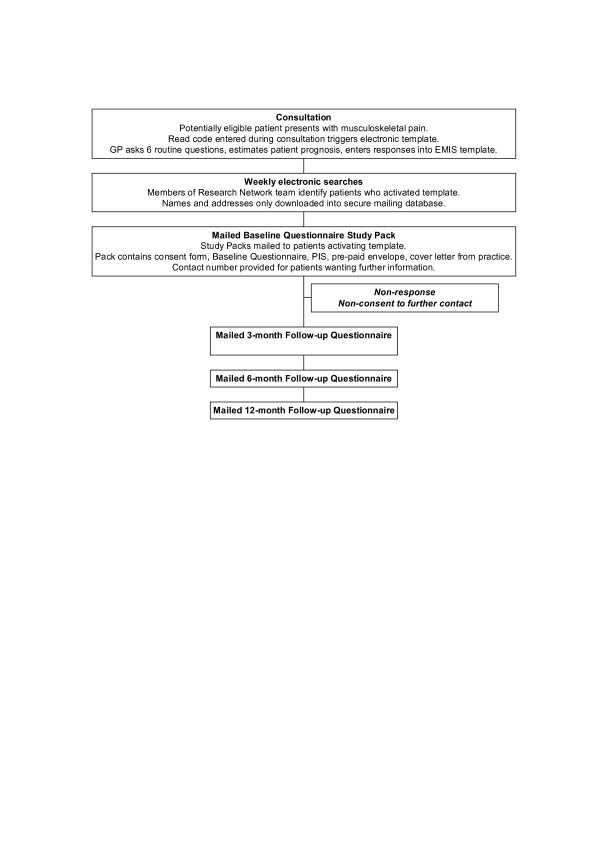
Flowchart of the recruitment process.

A flowchart of the process of recruitment is provided in Figure 2.

### Data collection

#### Brief prognostic assessment in the consultation

The primary prognostic data in this study are the six brief questions asked of the patient by the GP and the GP's prognostic judgement at the index consultation. The conceptual domains and corresponding operational definitions and empirical measures are listed below in Table 1.

#### Medical record review

In addition to accessing the responses to the brief prognostic assessment, medical records of consenting participants will be accessed to obtain the following information:

• Date of index consultation

• General Practice

• Index Read code

### Self-complete postal questionnaires

The other source of data collection in the study is self-complete postal questionnaires. This provides information on the descriptive characteristics of participants, outcome measurement, and more detailed prognostic information to supplement that collected from the brief prognostic assessment in the consultation. The conceptual domains, operational definitions, and empirical measures are provided below in Table 2.

### Data Entry, Coding, Cleaning, and Storage

Data will be entered into a database specifically designed for this study. Prior to data entry, this database will be tested using a set of dummy data. Data will be entered as the completed questionnaires are received by dedicated members of the administration team. Although they are experienced in data entry, specific training will be provided for this study. The index site of pain triggering the initial consultation will be entered into this database, so that subsequent mailings can refer to this index pain.

The lead investigator and the study statistician will determine coding prior to data entry. The database will provide coding options, to facilitate the entry of data. Some standard codes (e.g. missing data (-9), not applicable (-88)) are used by the Centre and will be utilised in this study.

A different member of the team will then check 1 in 10 random questionnaires as part of a quality assessment process. This information is kept by the research support co-ordinator and the study statistician.

Data will be stored in a password protected database. Only members of the research team will have access to the data. Requests for access to the data must be made in writing, along with an analysis plan, to the custodian of the data. Questionnaires and consent sheets are securely stored in separate locations, to protect the confidentiality of participants.

### Sample size

Using data from the 1991 National Survey of Morbidity in General Practice and more recent data from the North Staffordshire Primary Care Research Consortium's database of consultations, we estimate that the frequency of consultation for musculoskeletal conditions in those aged 50 years and over is 7 per 1000 registered patients per month (discounting repeat consultations within the same year). The approximate practice denominator population in this age-group for the 6 participating practices is 18,000. This means that the potential numbers of recruited patients over a 3 month period would be approximately 380. However we have to allow for a combined non-response and non-consent to medical record review of 20%. This will leave an estimated 300 participants completing 12-month follow up.

### Statistical Analysis

Data from the three sources (brief assessment tool, general practice medical records and self-complete questionnaire) will be analysed as follow:

I. Descriptive account of flow of participants: eligible, mailed, responded, consented, followed up

II. Descriptive account of completeness of data and comparison between brief prognostic assessment and self-complete versions of items

III. Descriptive characteristics of participants, including comparison between completers and non-completers.

IV. Description of frequency of recovery and non-recovery at 3, 6, and 12 months among completers.

V. Univariable comparison between GP prognostic judgement at baseline and recovery at 6 month follow up (Cox regression).

VI. Univariable comparison between patient prognostic judgement and recovery at 6 months (Cox regression).

VII. Univariable relation between brief prognostic assessment variables and recovery at 3, 6 and 12 months (Cox regression).

VIII. Multivariable relation between brief prognostic assessment variables and recovery at 6 and 12 months (Cox regression).

IX. Comparison of prognostic accuracy of GP judgement, patient judgement, and brief prognostic assessment for predicting recovery at 6 months

X. The marginal informativeness of brief prognostic assessment variables to GP prognostic judgement for predicting recovery at 6 months (multivariable Cox regression).

## Discussion

Prognosis is considered to be a fundamental component of scientific medicine [20] yet prognostic research in primary care settings is currently neglected [21] and prognostic enquiry is disappearing from general medical textbooks [22]. This study aims to address this issue by examining the use of generic prognostic factors in a general practice setting.

Patients aged over 50 years presenting to six general practices with selected musculoskeletal conditions will be asked a standardised set of six questions by their general practitioner. Participants will be followed up with a postal questionnaire at baseline, three, six and 12 months and by medical record review (for those who give consent).

Data gathered during the consultation will be used to see if generic prognostic indicators assessed at the time of initial consultation can estimate outcome at six and 12 months.

## Conflict of interests

The author(s) declare that they have no competing interests.

## Authors' contributions

All authors participated in the design of the study. CM and GP drafted the manuscript which was approved by all authors.

## Pre-publication history

The pre-publication history for this paper can be accessed here:



## Figures and Tables

**Table 1:** Brief prognostic assessment in the consultation. Conceptual domains, operational definitions, and empirical measures

Conceptual domain	Operational definition	Empirical measure	No. of items
Pain intensity	Current pain intensity (of the index pain, at the time of index consultation)	0–10 NRS [8]	1
Episode duration	Time since last whole month free from this pain	Episode duration [9]	1
Multiple site/widespread pain	Current pain in sites other than the index site in the last month	Yes/no	1
Pain interference with activities	Pain interference with daily activities in the past month (of the index pain)	0–10 NRS [8]	1
Depression	Low mood, anhedonia in the past month	Depression Screening questions for primary care [10]	2
GP Prognostic Judgement	GP predicted outcome at 6 months	6 item scale (completely recovered through to much worse) [adapted from 11]	1

**Table 2:** Self-complete postal questionnaires. Conceptual domains, operational definitions, and empirical measures

Conceptual domain	Operational definition	Empirical measure	No. of items	When
Index pain location	Site of index pain complaint	Choice of anatomical site	1	B
Index global severity	Composite characteristic pain intensity, interference with activities, and disability days (of the index pain, in the month preceding index consultation)	Chronic Pain Grade [8] †	7	B, 3FU, 6FU, 12FU
Nature of onset	Traumatic, atraumatic onset	Yes/no/unsure	1	B
Episode duration	Time since last whole month free from this pain	Episode duration [9] †	1	B, 3FU. 6FU, 12FU
Multiple site/widespread pain	Pain in sites other than the index site over the last month. Manchester definition of widespread pain; Keele definition of number of pain sites	Manikin [12, 13] †	1	B, 3FU, 6FU, 12 FU
Pain consultation	First consultation for this pain complaint	Yes/no	1	B
Origin pain	Cause of pain	Free text	1	B
Consultation reason	Reason for consulting GP for this pain	Free text	1	B
Prognostic discussion	Does patient recall GP discussing prognosis	Yes/no	1	B
Prognostic importance	Is discussing prognosis important to the patient	Yes/no with free text section for explanation	3	B
Anxiety	Anxiety symptoms in past week	HAD [14]	7	B, 3FU, 6FU, 12FU
Depression	Low mood, anhedonia in past week	HAD [14]	7	B, 3FU, 6FU 12FU
	Low mood, anhedonia in the past month	Depression Screening questions for primary care [10] †	2	B, 3FU, 6FU, 12FU
Coping strategies	Coping strategies questionnaire	1-item CSQ [15]	8	B
Patient Prognostic Judgement	Anticipated global change at 6 months	6 item scale (completely recovered through to much worse) (adapted from [11]	1	B
Patient Perceived Recovery	Patients self-perceived rating of recovery	Yes/no [11]	1	3FU, 6FU, 12FU
Age	Age at index consultation	Date of birth	1	B, 3FU, 6FU, 12FU
Sex	Sex	Male/Female	1	B, 3FU, 6FU, 12FU
Employment status	Employment status at time of questionnaire	Employed/Not working due to ill health/Retired/Housewife/Unemployed/Other	1	B,6FU. 12FU
Socioeconomic status	Occupational class based on individual (i) current or (ii) most recent job title	Job title – categorised as manual/non-manual according to SOCC 2000 [16, 17]	2	B
Living arrangement	Live alone	Yes/No	1	B
Marital status	Marital status at time of Baseline Questionnaire	Married/Single/Divorced/Widowed/Separated/Cohabiting	1	B
Social support	Availability of instrumental support	Single items [18] (yes/no/no need)	1	B, 3FU, 6 FU, 12 FU
	Availability of emotional support	(yes/no/no need)	1	
Obesity	Body Mass Index	Height (m/ft, in), weight (kg/st, lb)	2	B
General health	Self-rated health	SF-36 item [19]	1	B, 3FU, 6 FU, 12 FU

B = Baseline Questionnaire; 3FU = 3-month Follow-up Questionnaire HAD = Hospital Anxiety and Depression scale; SF-36 = Medical Outcomes Study Short-Form 36-item health status instrument

† Contains duplicate of brief prognostic assessment question
